# Woodhouse-sakati syndrome with no reportable MRI findings: a case report

**DOI:** 10.1186/s12883-024-03865-z

**Published:** 2024-09-28

**Authors:** Rebecca Eilish Irvine, Arshia Ahmad

**Affiliations:** 1 School of Medicine, Imperial College London, Exhibition Road, South Kensington, London, UK; 2 Wellington Hospital, HCA Healthcare, 27 Circus Road, London, UK

**Keywords:** Woodhouse-Sakati syndrome (WSS), MRI findings, Dystonia, Movement disorders, Deep brain stimulation (DBS), Neurorehabilitation

## Abstract

**Background:**

Woodhouse-Sakati Syndrome (WSS) is a rare autosomal recessive condition caused by biallelic pathogenic variants in the *DCAF17* gene, with fewer than 200 cases reported in the literature. Symptoms first emerge in middle-late adolescence with a spectrum of hypogonadal and progressive neurological features.

**Case presentation:**

We present a case of WSS with no reportable T_2_-weighted, apparent diffusion coefficient mapping and susceptibility weighted MRI findings. This differs from cases reported in the current literature. Our patient developed abnormal movements in both legs, clumsiness of the hands, dysarthria, and swallowing difficulties. Moreover, she presented with alopecia manifesting as frontal and temporal balding, severe dystonia with painful dystonic spasms primarily in the left upper limb, as well as primary amenorrhea. She was not independently ambulatory on presentation, requiring wheelchair assistance. Genetic testing, the crucial test for a definitive diagnosis, was undertaken in Qatar and confirmed WSS. Treatment provided includes botulinum toxin injections and deep brain stimulation, providing better dystonia control, with progress in walking and strength exercises, and overall remarkable improvement. Intensive neurorehabilitation regimes were also deployed from admission, including physiotherapy, occupational therapy and speech and language therapy.

**Conclusion:**

This case adds to the current literature on WSS manifestations, with all previously reported cases having positive MRI findings, unlike our case.

## Introduction

Woodhouse-Sakati Syndrome (WSS) is a rare autosomal recessive condition caused by biallelic pathogenic variants in the *DCAF17* gene, with fewer than 200 cases reported in the literature [1, 2]. Symptoms first emerge in middle-late adolescence with a spectrum of hypogonadal and progressive neurological features.

WSS was initially reported in the literature in 1983 by Nicholas J Y Woodhouse and Nadia A Sakati [3]. They observed six Saudi Arabian patients from two consanguineous families, and noted a ‘syndrome of hypogonadism, alopecia, diabetes mellitus, mental retardation, deafness, and ECG abnormalities’ [3].

WSS is commonly reported amongst consanguineous families, particularly of Middle Eastern descent. The underlying mechanisms of Woodhouse-Sakati Syndrome remain unknown; however, the regional preponderance of Middle Eastern countries may suggest an underlying founder effect [4]. The clinical heterogeneity of WSS in Qatar can be explained by the founder variant c.436delC in the *DCAF17* gene [2].

The purpose of this report is to show a case of WSS without MRI findings, but with clear phenotypical features. This is significant as all the cases of WSS reported in the literature have associated MRI findings [1, 5].

## Case report

### History, examinations & investigations

We present a case of Woodhouse-Sakati Syndrome in a 22-year-old female from Qatar. She had normal development, with onset of symptoms in late adolescence, and gradual worsening of symptoms over the last few years prior to the latest hospital admission. She developed abnormal movements in both legs, clumsiness of the hands, dysarthria, and swallowing difficulties. Moreover, she presented with alopecia manifesting as frontal and temporal balding (the crown of head is spared), severe dystonia with painful dystonic spasms, as well as primary amenorrhea as a result of hypogonadism. She also presents with type 2 diabetes mellitus, and a painful breast lump most likely hypogonadic in nature.

Moreover, she recently presented with ear pain bilaterally (more on the right-hand side). No findings were noted on otoscopy. ENT examination confirmed sensorineural hearing loss.

Her siblings (6 or 7 in number) also share intellectual disabilities, phenotypical facial features and alopecia features, but not the dystonia as seen in our patient. They have not yet undergone genetic testing for WSS, however.

**Fig. 1:**
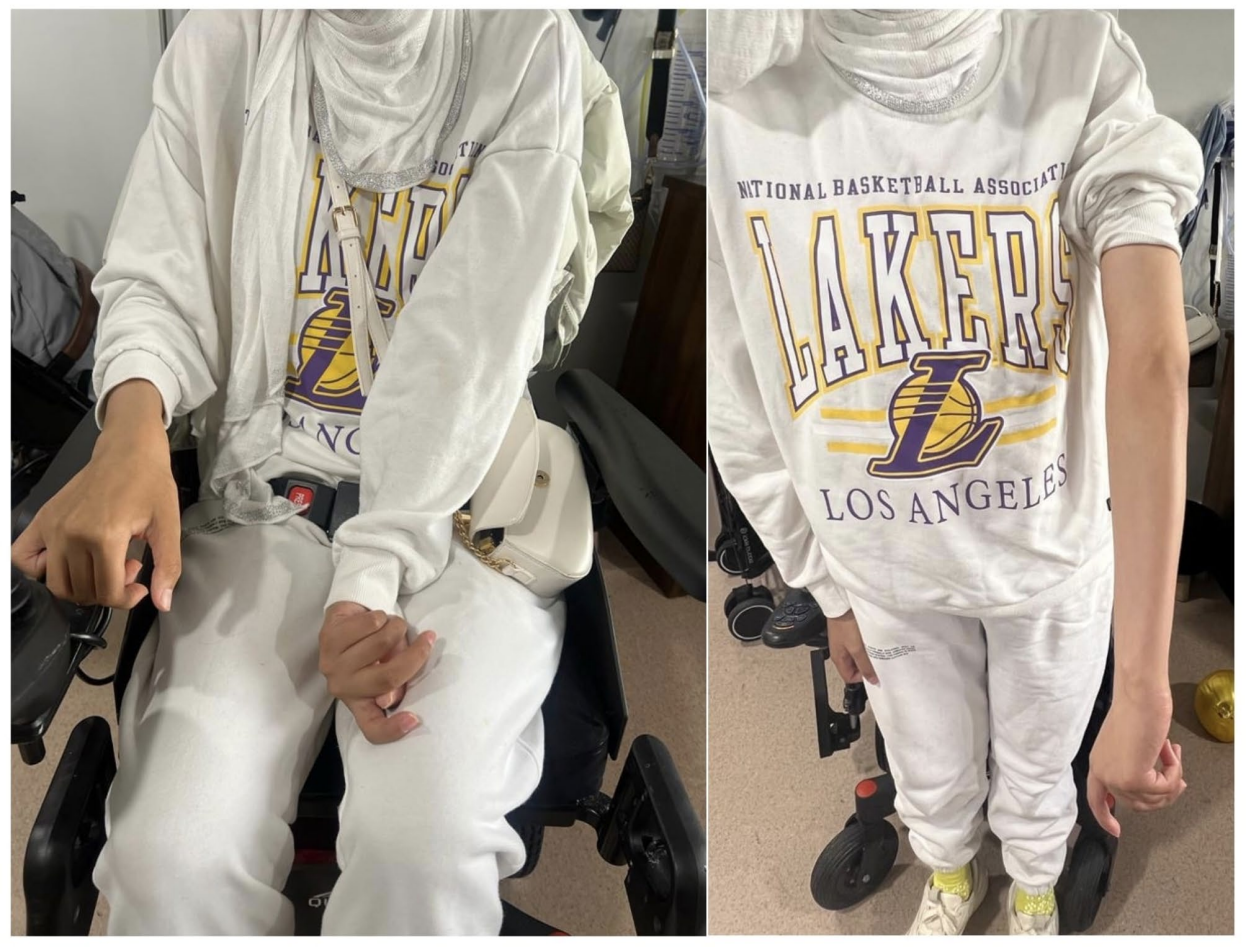
Dystonic posturing of fixed internal rotation of the left shoulder and flexion of the left elbow

On examination, she mobilises using a wheelchair. She has dystonic posturing including fixed internal rotation of the left shoulder, and flexion of the left elbow (Fig. 1). She has a female phenotype but poor secondary sexual development. Examination also revealed marked cognitive impairment.

**Fig. 2:**
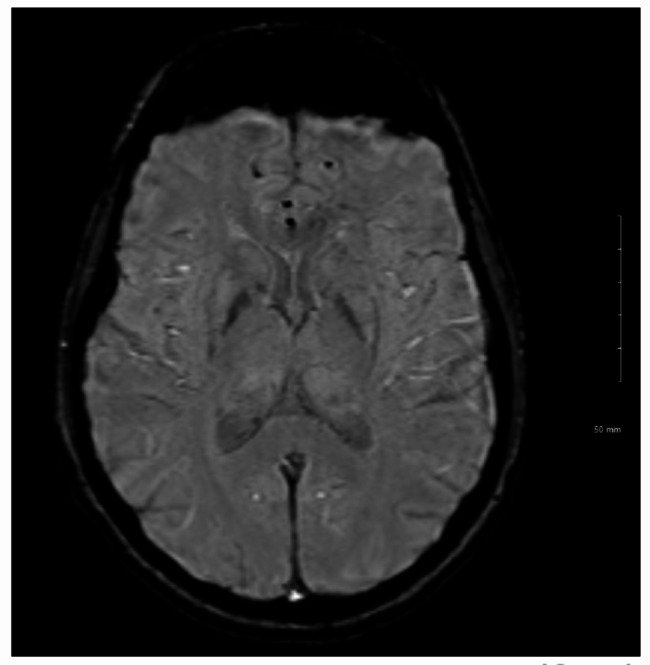
Axial susceptibility-weighted (SWI) MRI scan

**Fig. 3:**
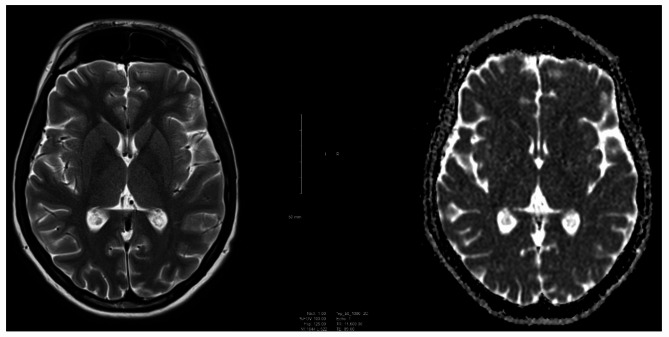
Axial T_2_-weighted (left) vs. apparent diffusion coefficient (ADC) map (right) MRI scans

Genetic testing undertaken in Qatar confirmed WSS, via sequence analysis of *DCAF17* with identification of biallelic pathogenic variants. T_2_-weighted, apparent diffusion coefficient mapping and susceptibility weighted MRI scans of the brain and spinal cord performed were unremarkable (Figs. 2 and 3).

Moreover, a pelvic ultrasound was performed, with the indication of primary amenorrhea, revealing a small uterus (measuring 4 cm in length, and 1.5 × 2.4 cm in cross section) and small ovaries (right ovary measuring 0.9 × 0.7 × 1.3 cm and left ovary measuring 1.6 × 2.55 × 0.9 cm).

Serum iron was measured at 16.1 umol/l (10.74–30.43 umol/l), and unsaturated iron-binding capacity (UIBC) was 23.8 umol/l (24.2–70 umol/l). Copper was normal, at 18.8 umol/l (12–25 umol/l), and ceruloplasmin was normal at 0.26 g/l (0.2–0.6 g/l).

### Treatment & progress

There is no specific treatment for WSS; treatment is aimed at relieving symptoms. As for most movement disorders, only a small range of symptomatic treatments are available [6]. A ‘trial and error’ approach to symptomatic treatment of the disorder is often deployed, as was the case with our patient.

Botulinum toxin injections, which have provided a remarkable advance in the treatment of those with focal dystonia [6], were given to our patient, to reduce tightness in the left pectoral region. And, subsequently were given in the adductor region of the left deltoid muscle, in an attempt to reduce ongoing painful spasms.

The current knowledge is that deep brain stimulation (DBS) is modestly effective in treating rare inherited dystonias with a combined phenotype [7]. DBS was trialled in our patient, which provided some immediate effect prior to it being switched on. Immediately following DBS insertion, spasms and dystonia were better controlled, and she appeared to be in less pain. There is still significant dystonia present, however it is much better, and she is able to walk more distance with support (approximately 20 m with support, compared to not being able to walk prior to DBS insertion and neurorehabilitation regimes). A month post-implantation, the device was switched on and provided better dystonia control, with progress in walking and strength exercises and overall remarkable improvement.

Intensive neurorehabilitation regimes were deployed since admission, and include physiotherapy, occupational therapy and speech and language therapy. These have proven to improve her speech and swallow, standing, and functional tasks (including grooming and dressing) with decreased involuntary dystonia during these tasks. There have also been improvements noted in handwriting, static balance (currently able to tolerate standing for 3 min) and improved exercise tolerance.

## Discussion

### Literature review & conclusion

Woodhouse-Sakati syndrome is a progressive extrapyramidal syndrome characterized by dystonia, dysarthria, cognitive decline, and endocrine abnormalities such as hypogonadism, alopecia, and diabetes mellitus. It follows an autosomal-recessive inheritance pattern and is caused by biallelic mutations in the DDB1 and CUL4 associated factor 17 (*DCAF17*) gene [8].

We reported a case of Woodhouse-Sakati Syndrome in a 22-year-old female from Qatar. The most salient features in this case were primary amenorrhea, alopecia, absence of secondary sexual characteristics, and significant dystonia.

Reviewing the currently available literature on WSS, diabetes mellitus, either the insulin-dependent (type I) or -independent (type II) form with onset in adolescence or early adulthood, was present in 60% of cases [4] including ours.

From a neurological point of view, more than half of the cases have progressive extrapyramidal movements such as dystonic spasms with dystonic posturing, dysarthria and dysphagia, moderate bilateral post-lingual sensorineural hearing loss, and mild intellectual disability [2].

Contrasting this, a literature review of 58 patients from Qatar concluded that extrapyramidal manifestations were found to be uncommon (8.6%). Ectodermal and endocrine (primary hypogonadism) manifestations were the most common presentations (100%), followed by diabetes mellitus (46%) and hypothyroidism (36%). Neurological manifestations were overlapping among patients with intellectual disability (ID) being the most common (75%). Moreover, distinctive facial features were noted in all patients [2].

In another study, a total of 38 individuals belonging to 17 families were identified to have WSS. The mean age at enrolment was 30.1 years (range 16–53 years). Neurological involvement was noted in 31 patients (81.5%). Dystonia was the most common neurological manifestation (67%), followed by intellectual disability (45%) and sensorineural hearing loss (30%) [9].

Although our patient did not initially present with sensorineural hearing loss, she subsequently developed bilateral ear pain, which was later diagnosed as sensorineural hearing loss following an ENT review. She did not present with hypothyroidism.

Common MRI findings amongst WSS patients include a small pituitary gland (76.9%), pronounced basal ganglia iron deposition (73%), and white matter lesions in 69.2% of patients, showing frontoparietal and periventricular predominance [5]. In a review of brain MRI images of 26 patients, all patients had abnormal MRI findings [5]. Moreover, a case report of two WSS patients revealed positive MRI findings in both patients [1].

This is in contrast with the MRI findings reported from our patient. Despite it being found that older age being associated with a more severe degree of white matter lesions (*P* < .001) [5], patients younger than ours were reviewed in this study (12 males, 14 females; age range, 16–45 years; mean age, 26.6 years).

A potential limitation with our case, is that due to the fact that she initially presented in Qatar, we cannot be certain as to whether there were MRI findings at initial presentation, albeit very unlikely. The patient will likely be under follow-up for several years at our institution; however, there is currently no plan to repeat an MRI analysis unless clinically indicated. It may be considered to repeat an MRI in 3–5 years to assess interval changes. In the current literature, the expansion of white matter lesions and further iron deposition during follow-up has been reported amongst patients with MRI findings present [5]. However, there is no report of a patient with a normal MRI scan at admission, as seen in our patient, later developing MRI findings during follow-up.

Overall, our case provides a novel report of a patient with genetically confirmed WSS with an array of pathognomonic symptoms, including significant dystonia, but with negative MRI findings. This presentation of negative MRI findings has not been previously reported in the existing literature, hence adding to the available literature on WSS manifestations.

## Data Availability

Data sharing is not applicable to this article as no datasets were generated or analysed during the current study.

## Abbreviations

WSS: Woodhouse-Sakati Syndrome
DCAF17: DDB1 and CUL4 associated factor 17
MRI: Magnetic resonance imaging
ENT: Ear, nose and throat
SWI: Susceptibility-weighted imaging
ADC: Apparent diffusion coefficient
UBIC: Unsaturated iron-binding capacity
DBS: Deep brain stimulation
ID: Intellectual disability
